# Allelic diversity of merozoite surface protein genes (*msp1* and *msp2*) and clinical manifestations of *Plasmodium falciparum* malaria cases in Aceh, Indonesia

**DOI:** 10.1186/s12936-021-03719-w

**Published:** 2021-04-13

**Authors:** Kurnia Fitri Jamil, Nandha Rizki Pratama, Sylvia Sance Marantina, Harapan Harapan, Muhammad Riza Kurniawan, Tjut Mariam Zanaria, Jontari Hutagalung, Ismail Ekoprayitno Rozi, Puji Budi Setia Asih, Supargiyono, Din Syafruddin

**Affiliations:** 1 Division of Tropical Medicine & Infectious Disease, Department of Internal Medicine Faculty of Medicine, Universitas Syiah Kuala, Banda Aceh, Indonesia; 2 Malaria and Vector Resistance Unit, Eijkman Institute for Molecular Biology, Jakarta, Indonesia; 3 Medical Research Unit, Faculty of Medicine, Universitas Syiah Kuala, Banda Aceh, Indonesia; 4 Recident of Internal Medicine Education Specialist Program Faculty of Medicine, Universitas Syiah Kuala, Banda Aceh, Indonesia; 5 Department of Parasitology School of Medicine, Universitas Syiah Kuala, Banda Aceh, Indonesia; 6 National Institute of Health Research and Development (NIHRD), Ministry of Health, Jakarta, Indonesia; 7 Center for Tropical Medicine/Department of Parasitology, Faculty of Medicine, Gadjah Mada University, Yogyakarta, Indonesia; 8 Department of Parasitology, Faculty of Medicine, Hasanuddin University, Makassar, Indonesia

**Keywords:** Allele, Malaria, *msp1*, *msp2*, *Plasmodium falciparum*, Clinical manifestations

## Abstract

**Background:**

The malaria control programme in Indonesia has successfully brought down malaria incidence in many parts in Indonesia, including Aceh Province. Clinical manifestation of reported malaria cases in Aceh varied widely from asymptomatic, mild uncomplicated to severe and fatal complications. The present study aims to explore the allelic diversity of merozoite surface protein 1 gene (*msp1*) and *msp2* among the *Plasmodium falciparum* isolates in Aceh Province and to determine their potential correlation with the severity of malaria clinical manifestation.

**Methods:**

Screening of over 500 malaria cases admitted to the hospitals in 11 districts hospital within Aceh Province during 2013–2015, identified 90 cases of *P. falciparum* mono-infection without any co-morbidity. The subjects were clinically phenotyped and parasite DNA was extracted and polymerase chain reaction (PCR) amplified for the *msp1* and *msp2* allelic subfamilies.

**Results:**

Analysis of clinical manifestation revealed that fever-chill is the most frequent symptom. Based on WHO criteria showed 19 cases were classified as severe and 71 as mild malaria. Analysis of *msp1* gene revealed the presence of K1 allele subfamily in 34 subjects, MAD20 in 42 subjects, RO33 in 1 subject, and mixed allelic of K1 + MAD20 in 5 subjects, K1 + RO33 in 4 subjects, and MAD20 + RO33 in 4 subjects. Analysis of *msp2* gene revealed 34 subjects carried the FC27 allelic subfamily, 37 subjects carried the 3D7 and 19 subjects carried the mixed FC27 + 3D7. Analysis of multiplicity of infection revealed that *msp1* alleles is slightly higher than *msp2* with the mean of MOI were 2.69 and 2.27, respectively. Statistical analysis to determine the association between each clinical manifestation and *msp1* and *msp2* alleles revealed that liver function abnormal value was associated with the *msp2* mixed alleles (odds ratio (OR):0.13; 95%CI: 0.03–0.53). Mixed *msp1* of K1 + RO33 was associated with severe malaria (OR: 28.50; 95%CI: 1.59–1532.30).

**Conclusion:**

This study found a strong association between severe malaria in Aceh with subjects carrying the *msp1* mixed alleles of K1 and RO33. The liver function abnormal value associated with the *msp2* mixed allelic subfamilies. Further study in different geographic areas is recommended.

## Background

Malaria remains a major public health problem in Indonesia despite a success in bringing down the incidence within the last few decades. Since the implementation of the malaria elimination programme in 2009, the malaria cases in Indonesia dropped significantly from 418,439 to 261,617 and the vast majority of the cases occurred in 5 provinces of the eastern parts of country [1]. In Aceh Province, effective monitoring of the implementation of malaria elimination programme has also successfully brought down the annual parasite incidence (API) from 0.08 in 2015 to 0.06 in 2017. The success of malaria control programme relies on three main efforts: early diagnosis and prompt treatment, provision of long-lasting insecticidal bed nets (LLINs) and insecticide residual spraying. Attempts to develop a suitable vaccine to prevent malaria were so far fruitless as several vaccine candidates produced still fail to meet the required efficacy [2].

The clinical manifestation of malaria infection in human varies widely, from asymptomatic to fatal infections with cerebral or non-cerebral complications. This phenomenon is associated with factors associated with the malarial parasite, human host and environment. Studies to identify parasite factors that contribute to virulence revealed several candidates such as antimalarial drug resistance, cytoadherence, and antigenic polymorphisms [3]. Clinical manifestation of malaria is directly associated with the repeated cycle of invasion of the red blood cell (RBC) by merozoites, followed by development into schizont which end up in the rupture of the RBC and release of daughter merozoites. During the blood stage, the parasite expresses arrays of proteins and among others are merozoite surface protein 1 (MSP1) and MSP2. These proteins are involved in erythrocyte invasion [4] and are targeted by the immune responses [5, 6], and therefore have been used as target for vaccine development. The *msp1* and *msp2* genes also exhibit high polymorphisms hence play important role in identification of genetically distinct *P. falciparum* parasite sub-populations [4]. The *msp1* gene is located on chromosome 9 and contains 17 blocks of sequences [7]; block 2 is most polymorphic and is grouped into three allelic families MAD20, K1 and RO33 [8]. The *msp2* gene is located on chromosome 2 composed of 5 blocks and block 3 is the most polymorphic [9]. The *msp1* alleles are grouped into two allelic families, FC27 and IC1/3D7) [10].

Many studies have explored the potential roles of *msp1* and *msp2* alleles in the modulation of malaria clinical manifestations. In French Guyana, study on the *msp1* and *var* genes demonstrated that *msp1* K1 allele and *var* genes, D allele overexpression associated with severe malaria [11]. Multiple field studies have tried to characterize virulent strain of *P. falciparum* using genetic polymorphisms as markers. Although evidence of differences in virulence among the *P. falciparum* strains have accumulated, the virulent strains have not yet been characterized in sufficient detail to identify suitable virulence markers. The present study aims to explore the allelic diversity of merozoite surface protein genes among the *P. falciparum* isolates in Aceh Province and their potential association with the severity of clinical manifestation of malaria.

## Methods

### Ethical statement

This study has been approved by the Medical and Health Research Ethic Committee, Faculty of Medicine Gajah Mada University, with reference No: KE/FK/173/EC. All subjects were asked for informed consent prior to participation.

### Study site

**Fig. 1:**
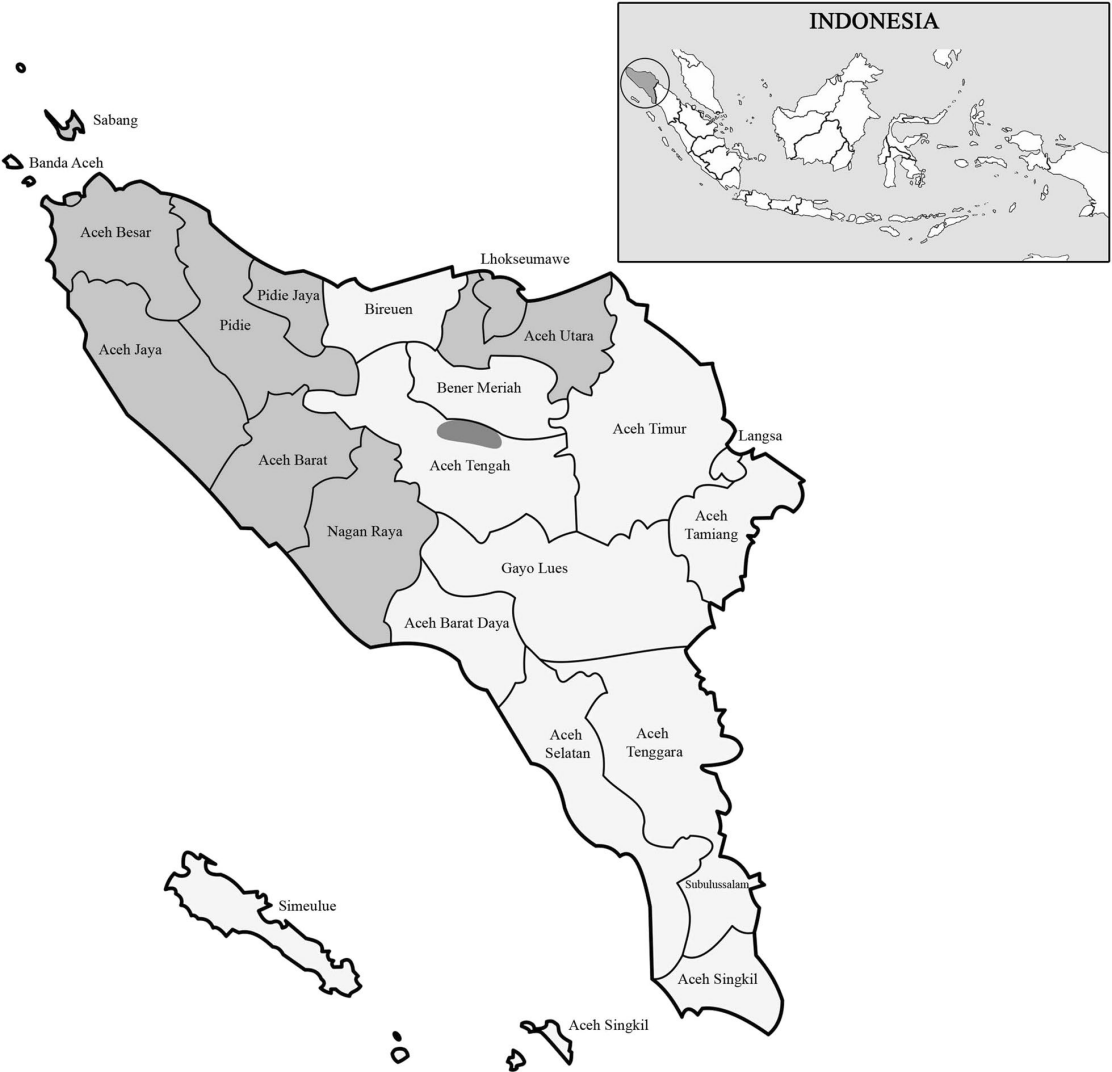
Study sites in Aceh; Kota Banda Aceh, Sabang, Kota Lhokseumawe, Aceh Besar, Aceh Barat Daya, Nagan Raya, Aceh Barat, Aceh Jaya, Aceh Utara, Pidie Jaya, and Pidie

**Fig. 2:**
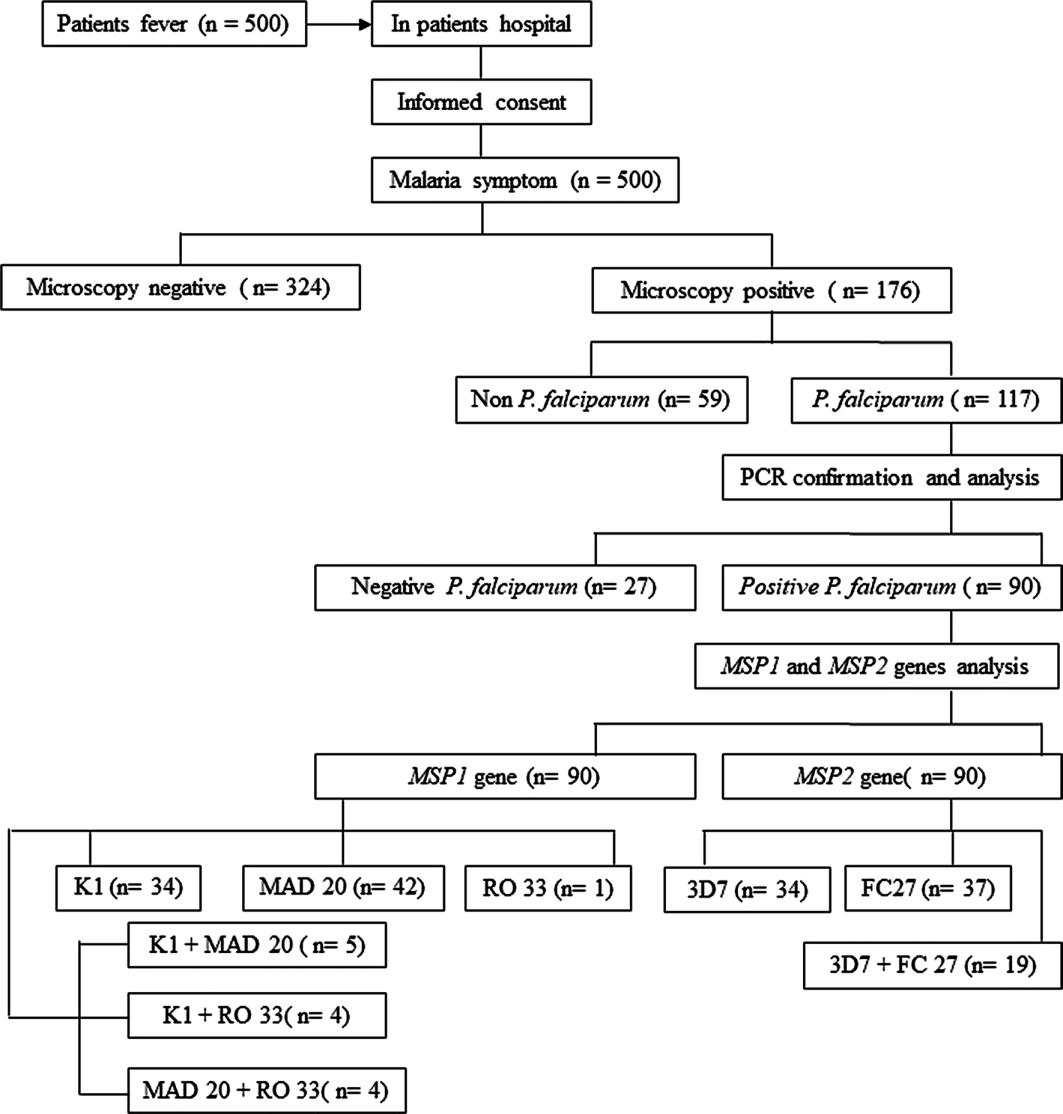
Study flowchart samples collection

This study was conducted in eleven district hospitals in Aceh Province, Indonesia (Fig. 1). Patients admitted with initials diagnosis of malaria were screened for cases with mono infection of *P. falciparum* without any co-morbidity (Fig. 2). At admission, thick and thin blood films were obtained using finger prick, stained with Giemsa and examined by accredited microscopists. Any subjects declared positive by microscopy will be further stated in parasite density per microlitre blood as previously described. Dried blood spots were made using 3MM Whatman 3 M paper (GE Healthcare, Buckinghamshire, UK) and kept in individual plastic ziplock until used.

### Determination of malaria severity

Upon admission to the hospital, all subjects underwent physical examination and laboratory assays for complete blood count, thin and thick blood smear, liver and kidney function tests. History taking and consent were obtained from patients or legal guardian. Clinical manifestation of the patients was assessed and classified using the World Health Organization (WHO) criteria [12].

### Criteria for severe malaria

Severe falciparum malaria is defined as one or more of the following [11], occurring in the absence of an identified alternative cause and in the presence of *P. falciparum* asexual parasitaemia: (a) impaired consciousness, (b) prostration, (c) multiple convulsions, (d) hypoglycaemia, (e) renal impairment, (f) jaundice, (g), pulmonary oedema and (h) significant bleeding, such as haematemesis or melaena.

### DNA extraction and polymerase chain reaction

The parasite DNA was extracted from the filter paper using Chelex-100 ion exchanger method as described previously [13]. The DNA extract was used as template for the nested-1 PCR for determining species of the parasite [14] and only cases with monoinfection with *P. falciparum* will be enrolled. All enrolled subjects will be PCR amplified using oligos that target the parasite *msp1* [M1-OF 5′- CTAGAAGCTTTAGAAGATGCAGTATTG -3′ and M1-OR 5′- CTTAAATAGTATTCTAATTCAAGTGGATCA -3′] and *msp2* genes [M2-OF 5′- ATGAAGGTAATTAAAACATTGTCTATTATA -3′ and M2-OR 5′- CTTTGTTACCATCGGTACATTCTT -3′] with a total of 25 µl volume was used for all reactions [15]. Furthermore, primers for the second amplification reaction (Nested 2) were used following the procedure described previously: primer set targets specific allelic families of *msp1* (MAD20, K1 and R033), and *msp2* (IC/3D7 and FC27); K1 = F-5: AAA-TGA-AGA-AGA-AAT-TAC-TAC-AAA-AGG-TGC-3 and R-5: GCT-TGC-ATC-AGC-TGG-AGG-GCT-TGC-ACC-AGA-3; MAD20 = F-5: AAA-TGA-AGG-AAG-AAC-TGG-AAC-AGC-TGT-TG-3 and R-5: ATC-TGA-AGG-ATT-TGT-ACG-TCT-TGA-ATT-ACC-3; RO33 = F-5: TAA-AGG-ATG-GAG-CAA-ATA-CTC-AGT-TGT-TG-3 and F-5: CAT-CTG-AAG-TTG-GAT-CAG-CAC-CTG-GAG-ATC-3; 3D7 = F-5: AGA-AGT-ATG-GCA-GAA-AGT-AAK-CCT-YCT-ACT-3 and R-5: GAT-TGT-AAT-TCG-GGG-GAT-TCA-GTT-TGT-TCG-3; FC 27 F-5: ACT-AAG AAT-AGT-GTA-GGT-GCA-RAT-GCT-CCT-3 and R-5: TTT-TAT-TTG-GTG-CAT-TGC-CAG-AAC-TTG-AAC-3F [15]. The second reaction primer set targets specific allelic families of *msp1* (MAD20, K1 and R033) or *msp2* (IC/3D7 and FC27). Reactions for each set of primary and nested primers were performed separately.

### Multiplicity of infection (MOI)

MOIs were calculated by dividing the total number of distinct *msp1* and *msp2* genotypes by the number of positive samples for each marker. The mean MOI was calculated by dividing the total number of alleles detected in both *msp1* and *msp2* by the total number of positive samples for both markers. Samples were considered single infected when harbouring only one allele at each of the genotyped loci. Multiclonal infections were defined as infections with more than one allele in at least one locus.

### Statistical analysis

All data were collected with Epidata and analysed by R version 3.4.0. The allelic subfamily frequency of *msp1* and *msp2* was calculated as the proportion of the allele detected for each allelic family out of the total of alleles detected. The frequency of polyclonal infection was calculated using number of samples with more than one amplified fragment out of the total samples. The mean MOI was determined by dividing the total number of alleles detected in both *msp1* and *msp2* by the total number of positive samples for both markers. The Chi-square test was used to analyse the association of the clinical manifestations and the allelic subfamilies of the *msp1* and *msp2*. Statistical significance was defined as p < 0.05.

## Results

The study subject’s recruitment flowchart and the procedures applied to subjects is shown in Fig. 2. Of the total 500 subjects admitted to the hospitals with fever, 176 subjects (52 males and 38 females) were found positive by microscopy and 117 of which by *P. falciparum*. Further validation by PCR revealed 90 subjects with *P. falciparum* mono infection. Among the 90 study subjects, 57.7% of them were males and 42.3% were females with most subjects 46.7% aged between 21 and 30 years old (Table 1).

**Table 1:** Characteristics of study subjects *Plasmodium falciparum* malaria cases

Variable	Overall
Number of patients enrolled	90
Age (year)	
Mean	33 (36%)
Range	19–63
< 20	6 (6.66%)
21–30	42 (46.7%)
31–40	23 (25.55%)
41–50	12 (13.23%)
51–60	5 (5.55%)
> 60	2 (2.22%)
Gender	
Male [n (%)]	52 (58%)
Female [n (%)]	38 (42%)
Body temperature [°C, mean (SD)]	
< 37.5 °C	0
≥ 37.5 °C	90 (100%)
Parasite density (/ul)	
Range	5000–15.000
Mean	12.500
> 10.000	82 (91%)
≤ 10.000	8 (9%)

### Clinical manifestation of the subjects

The clinical manifestation, origin and laboratory profiles of each subjects is shown in Table 2. The commonly observed symptoms and signs includes fever with chill (100%), dyspnoea (75.6%) and spleen enlargement (87.8%). Severe signs such as shock, convulsion and conscious disturbance were observed in few cases. Laboratory assays revealed anaemia in 36.8% of the subjects, abnormalities in the values of liver (63%) and kidney (95.6%) function, and haemoglobinuria (20%). Of the 90 subjects examined 92% had parasite density of 10,000 parasites per microlitre blood and the remaining 8% had parasite density of less than 10,000/µl blood. The parasite density of the subjects ranged from 5000 to 15,000 parasites per microlitre blood. Based on WHO classification [12], 19 (21%) subjects were classified as severe malaria whereas the remaining 71 (79%) subjects were mild, uncomplicated malaria.

**Table 2:** Clinical manifestation and laboratory profiles in Aceh Province

Profile	Symptoms and Signs	Total
Physical examination	Fever (≥ 37.5 °C)	90 (100*)
	Dyspnea	68 (75.6)
	Splenomegaly	79 (87.8)
	Convulsion	2 (2.2)
	Conscious disturbance	4 (4.4)
	Shock	3 (3.3)
Laboratory parameters	Anemia	33 (36.7)
	Abnormality in the values of liver function	57 (63.3)
	Abnormality in the values of kidney function	86 (95.6)
	Haemoglobinuria	18 (20)

(*) = percentage (%)

### Allelic subfamily frequency of the *msp1* and *msp2* genes

**Fig. 3:**
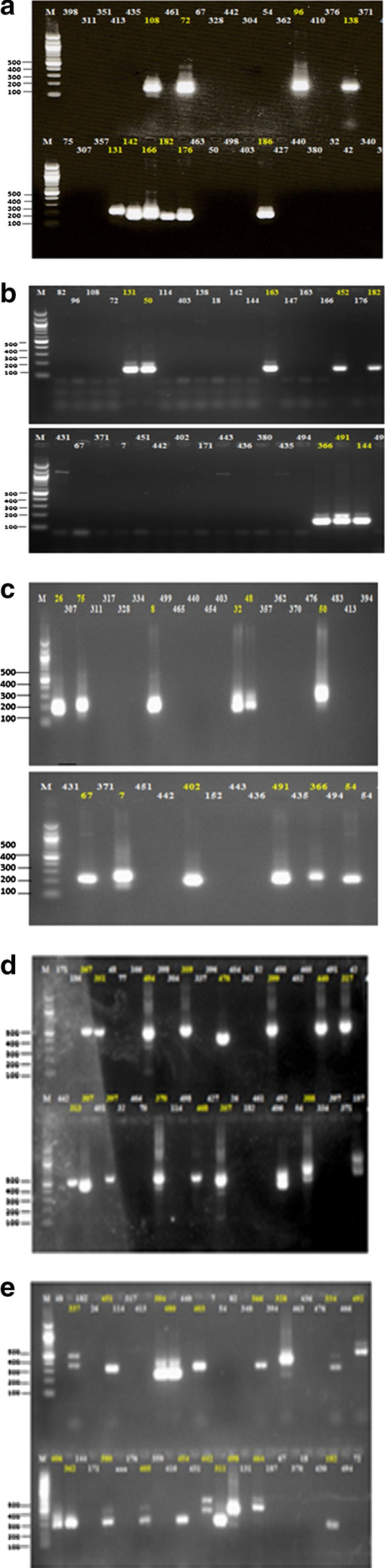
Electrophoregram of the *msp1* and *msp2* PCR are shown for the following pictures: **a** MAD20 (160–500 bp); **b** RO33 (130–220 bp); **c** K1 (160–350 bp); **d** 3D7 (100–450 bp); and **e** FC27 (250–530 bp)

Allelic analysis of the *msp1* gene revealed the existence of K1 (37.7%), MAD20 (46.7%), and RO33 (1.1%) subfamily, either as single or mixed allelic subfamilies. Mixed allelic subfamilies infections between K1 and MAD20, K1 and R033 and MAD20 with RO33 were also found in less frequency (Table 3). Analysis of the size of amplicons in each subfamily revealed that K1 subfamily had 3 alleles, MAD-20 had 5 alleles and RO33 had 2 alleles. Allelic analysis of the *msp2* gene revealed that there were two different allelic subfamilies. Of the total 90 subjects examined, the FC27 subfamily was observed in 41.4%, the 3D7 subfamily in 37.7% of the subjects and 21.2% of the subjects carried the mixed subfamilies of FC27 and 3D7. Analysis of the size of amplicons in each *msp2* subfamily revealed that FC27 had 4 alleles and 3D7 had 3 alleles (Table 3, Fig. 3).

**Table 3:** Allelic frequency of the *msp1* and *msp*2 genes in Aceh

Gene	Allele sub-family	Number	(%)	Amplicon	Allele
*msp1*	K1	34	37.7	160–350*	3
	MAD20	42	46.7	160–500	5
	RO33	1	1.1	130–220	2
	K1 + MAD20	5	5.6	–	–
	K1 + RO33	4	4.4	–	–
	MAD20 + RO33	4	4.4	–	–
*msp2*	FC27	37	41.1	250–530	4
	3D7	34	37.7	100–450	3
	FC27 + 3D7	19	21.2	–	–

*Base pairs

### Multiplicity of infection (MOI)

Of the total 90 subjects analysed for the allelic subfamilies of *msp1* and *msp2*, 13 subjects (14.4%) were found to carry multiple allelic subfamilies infection of *msp1*, whereas for *msp2*, 19 subjects (21.1%) were found. The MOIs for both *msp1* and *msp2* were 2.27 and 2.69, respectively. The MOI for *msp2* was slightly higher than *msp1* (Table 3). However, the amplicons were not run in a high concentration agarose gel to confirm the size of the amplicons.

### Association of clinical manifestation with the *msp1* and *msp2* alleles subfamilies

Analysis of *msp1* and *msp2* allelic subfamilies and each form of clinical manifestation and laboratory profiles revealed that abnormal liver function abnormal value was the only variable showing significant correlation with multiple allelic subfamilies of FC27 + 3D7 (OR: 0.13; 95%CI 0.03 to 0.53, p < 0.01). All others clinical manifestations showed no significant correlation with allelic subfamilies of *msp1* and *msp2* (p > 0.05) (Tables 4, 5). Based on the severity of clinical manifestation as classified by the WHO [11], subjects carried the mixed allelic subfamilies of K1 and RO33 of *msp1* had higher chance to have severe malaria (OR: 28.50; 95%CI: 1.59–1532.30) (Table 6). Other alleles revealed either no association or insignificant p-value.

**Table 4:** Association between clinical manifestation and allelic subfamilies

Clinical manifestation	Allele	Yes		No		p-value	OR	CI95%
		n	%	n	%			
Shock	K1	2	66.7	32	36.8	0.66	3.29	0.31–34.97
	MAD20	0	0	42	48.3	0.29	1.07	0.99–1.15
	RO33	0	0	1	1.1	1.00	1.03	1.00–1.08
	K1 + MAD20	0	0	5	5.7	1.00	1.04	1.00–1.08
	K1 + RO33	1	33.3	3	3.5	0.30	10.75	1.21–95.20
	MAD20 + RO33	0	0	4	4.6	1.00	1.04	1.00–1.08
Convulsion	K1	2	100	32	36.4	0.27	0.94	0.87–1.02
	MAD20	0	0	42	47.7	0.54	1.04	0.98–1.11
	RO33	0	0	1	1.1	1.00	1.02	0.99–1.06
	K1 + MAD20	0	0	5	5.7	1.00	1.02	0.99–1.06
	K1 + RO33	0	0	4	4.6	1.00	1.02	0.99–1.06
	MAD20 + RO33	0	0	4	4.6	1.00	1.02	0.99–1.06
Conscious disturbance	K1	2	50	32	37.2	1.00	1.65	0.24–11.16
	MAD20	0	0	42	48.8	0,16	1.09	1.00–1.19
	RO33	0	0	1	1.2	1,00	1.05	1.00–1.10
	K1 + MAD20	0	0	5	5.8	1.00	1.05	1.00–1.10
	K1 + RO33	1	25	3	3.5	0.42	7.17	0.94–54.51
	MAD20 + RO33	1	25	3	3.5	0.42	7.17	0.94–54.51
Anaemia	K1	14	42.4	20	35.1	0.64	1.21	0.71–2.09
	MAD20	17	51.5	25	43.9	0.63	1.21	0.71–2.09
	RO33	1	3	0	0	0.78	2.78	2.11–3.67
	K1 + MAD20	1	3.1	4	7	0.75	0.53	0.09–3.13
	K1 + RO33	0	0	4	7	0.31	1.62	1.37–1.92
	MAD20 + RO33	0	0	4	7	0.31	1.62	1.37–1.92
Haemoglobinuria	K1	9	50	25	34.7	0.36	1.65	0.73–3.74
	MAD20	6	33.3	36	50	0.32	0.57	0.24–1.39
	RO33	0	0	1	1.3	1.00	1.25	1.13–1.39
	K1 + MAD20	1	5.6	4	5.6	1.00	1.00	0.16–6.07
	K1 + RO33	2	11.1	2	2.8	0.37	2.69	0.92–7.87
	MAD20 + RO33	0	0	4	5.6	0.70	1.26	1.13–1.41
Splenomegaly	K1	29	36.7	5	45.4	0.82	0.96	0.81–1.13
	MAD20	38	48.1	4	36.4	0.68	1.06	0.91–1.23
	RO33	1	1.3	0	0	1.00	1.14	1.06–1.23
	K1 + MAD20	5	6.3	0	0	0.88	1.15	1.06–1.25
	K1 + RO33	3	3.8	1	9.1	0.99	0.85	0.48–1.50
	MAD20 + RO33	3	3.8	1	9.1	0.99	0.85	0.48–1.50
	MAD20	26	45.6	16	48.5	0.90	1.06	0.76–1.49
	RO33	1	1.8	0	0	1.00	1.68	1.41–1.99
	K1 + MAD20	1	7	1	3	0.16	0.32	0.06–1.87
	K1 + RO33	4	7	0	0	0.25	1.72	1.44–2.06
	MAD20 + RO33	2	3.5	2	6.1	1.00	0.83	0.31–2.24
Dyspnea	K1	26	38.3	8	36.4	1.00	1.00	0.79–1.26
	MAD20	33	48.5	9	40.9	0.88	1.05	0.83–1.31
	RO33	1	1.5	0	0	1.00	1.31	1.17–1.47
	K1 + MAD20	2	2.9	3	13.7	0.15	0.51	0.17–1.49
	K1 + RO33	3	4.4	1	4.5	0.86	0.81	0.05–47.66
	MAD20 + RO33	3	4.4	1	4.5	0.86	0.81	0.05–47.66
Abnormality in the value of kidney function	K1	32	37.2	2	50	–	–	–
	MAD20	40	46.5	2	50	–	–	–
	RO33	1	1.3	0	0	–	–	–
	K1 + MAD20	5	5.8	0	0	–	–	–
	K1 + RO33	4	4.6	0	0	–	–	–
	MAD20 + RO33	4	4.6	0	0	–	–	–
Fever (≥ 39.0 °C)	K1	23	44.2	11	28.9	0.22	1.84	0.6–5.66
	MAD20	17	32.7	25	65.8	–	–	–
	RO33	1	1.9	0	0	0.22	1.84	0.6–5.66
	K1 + MAD20	3	5.8	2	5.3	–	–	–
	K1 + RO33	4	7.7	0	0	–	–	–
	MAD20 + RO33	4	7.7	0	0	0.77	1.32	0.13–17.73

**Table 5:** Association between clinical manifestation and *msp2* allelic subfamilies

Clinical manifestation	Allele	Yes		No		p-value	OR	CI95%
		n	%	n	%			
Shock	3D7	1	33.3	36	41.4	1.00	0.72	0.07–7.61
	FC27	2	66.7	32	36.8	0.66	3.29	0.31–34.97
	FC27 + 3D7	0	0	19	21.8	0.85	1.04	0.99–1.10
Convulsion	3D7	1	50	36	40.9	1.00	1.43	0.09–22.18
	FC27	1	50	33	37.5	1.00	1.65	0.11–25.48
	FC27 + 3D7	0	0	19	21.6	1.00	1.03	0.99–1.07
Conscious disturbance	3D7	2	50	35	40.7	1.00	1.43	0.21–9.72
	FC27	1	25	33	38.4	0.99	0.55	0.06–5.07
	FC27 + 3D7	1	25	18	20.9	1.00	1.25	0.14–11.31
Anaemia	3D7	15	45.5	22	38.6	0.68	1.19	0.69–2.05
	FC27	10	30.3	24	41.2	0.38	0.72	0.39–1.32
	FC27 + 3D7	8	24.2	11	19.3	0.78	1.20	0.65–2.21
Haemoglobinuria	3D7	5	27.8	32	44.5	0.31	0.55	0.21–1.41
	FC27	11	61.1	23	31.9	0.04	2.59	1.11–6.03
	FC27 + 3D7	2	11.1	17	23.6	0.40	0.47	0.12–1.86
Splenomegaly	3D7	32	40.5	5	45.4	1.00	0.98	0.83–1.14
	FC27	31	39.2	3	27.3	0.66	1.06	0.92–1.24
	FC27 + 3D7	16	20.3	3	27.3	0.89	0.95	0.77–1.17
Abnormality in the value of liver function	3D7	27	47.4	10	30.3	0.17	1.29	0.95–1.75
	FC27	25	43.8	9	27.3	0.18	1.29	0.95–1.74
	FC27 + 3D7	5	8.8	14	42.4	0.00	1.36	0.17–0.77
Dyspnea	3D7	27	39.7	10	45.4	0.82	0.94	0.71–1.20
	FC27	28	41.2	6	27.3	0.36	1.15	0.92–1.45
	FC27 + 3D7	13	19.1	6	27.3	0.61	0.88	0.63–1.23
Abnormality in the value of kidney function	3D7	35	40.7	2	50	1.00	0.98	0.90–1.08
	FC27	33	38.4	1	25	0.99	1.03	0.94–1.12
	FC27 + 3D7	18	20.9	1	25	1.00	0.99	0.88–1.11
Fever (≥ 39.0 °C)	3D7	18	34.6	19	50	0.21	0.76	0.51–1.12
	FC27	24	46.2	10	26.3	0.09	1.41	1.00–1.98
	FC27 + 3D7	10	19.2	9	23.7	0.80	0.89	0.56–1.42

**Table 6:** Association of malaria severity with the *msp1* and *msp2* alleles

Gene	Allele	Severe		Mild		p-value	OR	CI95%
		n	%	n	%			
*msp1*	K1	7	36.8	27	38	0.17	2.46	0.55–12.50
	MAD20	4	21	38	53.6	–	–	–
	RO33	0	0	1	1.4	–	–	–
	K1 + MAD20	1	5.4	4	5.6	0.47	2.37	0.03–33.59
	K1 + RO33	3	15.8	1	1.4	0.00	28.50	1.59–15.32
	MAD20 + RO33	4	21	0	0	–	–	–
*msp2*	3D7	7	36.8	30	42.3	–	–	–
	FC27	11	57.9	23	32.4	0.19	2.04	0.6–7.22
	FC27 + 3D7	1	5.3	18	25.3	0.16	0.23	0.01–2.15

## Discussion

Analysis of the clinical manifestation of the malaria cases admitted to the hospitals in Aceh revealed that the classical symptoms, such as fever with chill is still the primary symptom experienced by the patients, followed by spleen enlargement and dyspnoea. Majority of the malaria cases are classified as mild, uncomplicated malaria but over 20% of which are severe according to WHO criteria [12]. The findings are in accordance with the report of the Ministry of Health where malaria in many parts of Aceh continues to decrease, making the risk to having malaria also diminishing and affect mainly adults who stay or travel to remaining endemic foci in the Province or other parts of the country [15]. This situation is completely different with the malaria cases found in eastern parts of the country where children are still the most vulnerable group and adults are usually asymptomatic [16, 17].

Analysis of the MOI based on the allelic diversity of *msp1* and *msp2* revealed a value of 2.27 and 2.67 for the *msp1* and *msp2*, respectively. This finding is slightly higher than the MOI data from Myanmar [18] and Southwest Pacific [19], but lower than Thailand, Kenya and Burkina Faso [20–23]. The difference in MOI can be attributed to several factors such as differences in geographical areas, intensity of malaria transmission, and difference in age of study population and mean parasite density in the study population [24–26]. In this study, all subjects are adult with symptomatic malaria and therefore with a relatively higher parasite density. Several studies reported conflicting results in which the MOI correlates with ages, parasite density [26, 27] and intensity of malaria transmission [28] but others studies failed to demonstrate this correlation [29, 30]. Low MOI reported in this study might also be attributed to the decreasing malaria transmission intensity in Aceh, following the implementation of the malaria elimination program in the area. It is of interest to note that despite a significant reduction of malaria in the area, a challenge of zoonotic malaria is currently increasing [31]. With regard to the parasite density, we did not observe any subjects with extremely high parasite density, such as parasite density > 100,000 per microlitre blood. This finding indicate that parasite density is not a contributing factor to specific clinical manifestation.

Analysis on the genetic diversity profiles of *P. falciparum* with the malaria clinical manifestation may provide useful information about parasite characteristics to design specific intervention strategies targeting the virulence factors [32] as well as to the evaluation of drug efficacy [33]. This study is the first study in Indonesia that provides information about genetic diversity of *msp1* and *msp2* alleles of *P. falciparum* among the hospitalized malaria cases. Analysis to determine the association between allelic subfamilies of the *msp1* and *msp2* with malaria severity revealed a strong association between mixed *msp1* K1 + RO33 with severe malaria. The finding is slightly different with that of Ariey et al*.* [10] where K1 allele subfamily and the D allele of *var* gene overexpression associated with severe malaria. Other findings reported a high proportion of subjects carrying the FC27 allele subfamily among the uncomplicated malaria patients [34]. This difference might be associated with the multifactorial nature of severe malaria with diverse clinical manifestation [2]. The difference in geographic setting, age of the subjects, and genetic background of the parasites and host certainly contribute substantial role. This finding is in accordance with the findings in previous studies in Bobo-Dioulasso [35]. Another study has also investigated genetic diversity of *P. falciparum* isolates, which was conducted in Libreville, Gabon. In the study, extensive genetic polymorphism within *msp1* allelic families (30 alleles identified) has been observed. This is consistent with the diversity found in Bakoumba (25 alleles) in 1999, Senegal (33 alleles) in 1995, and in Mauritania (27 alleles) in 2010 [36, 37]. This study on the distribution of *msp2* allele subfamilies showed the presence of two allelic subtypes in the study area, which is similar to the results of study conducted by Kang et al. in Myanmar where the geographical areas are alike. Their study also found only two allelic subtypes in Myanmar, which are the FC27 and 3D7. The difference between their study and our study is that the number of patients infected by mixed allelic subtypes is higher than patients affected by single infections; while our study has demonstrated contrasted finding where single infection, either by FC27 or 3D7 alleles is higher [37, 38]. This study also evaluated each clinical manifestations and symptoms among patients with falciparum malaria in Aceh Province and found out association between liver function abnormal value with mixed allelic *msp2* infection. This finding has never been analysed in previous studies except for the severe malarial anaemia [34]. Despite different results found, a closer observation on the phenotypes deserves further exploration in the other geographic areas. This study acknowledges several limitations such as the inadequate amount of sample size, difficulties to precisely estimate the allelic frequencies and genetic diversity due to the detection limit of the PCR technique used in the study. Alleles with short differences in length (less than 10 bp) might not be clearly distinguished.

## Conclusion

Allelic subfamilies analysis of the *msp1* and *msp2* genes among the hospitalized uncomplicated and severe malaria cases in Aceh have been analysed. Association between liver function abnormal value with the mixed allelic type of *msp2* was observed. Mixed allelic infection of *msp1* K1 and RO33 is strongly associated with severe malaria. This study has several limitations such as analyzing symptomatic malaria cases and only few severe cases. Further study to explore more subjects in different geographic setting and different clinical manifestation is recommended.

## Data Availability

All relevant data are within the manuscript.
